# A Cross-Linguistic Study of Individual Differences in Speech Planning

**DOI:** 10.3389/fpsyg.2021.655516

**Published:** 2021-05-07

**Authors:** Benjamin Swets, Susanne Fuchs, Jelena Krivokapić, Caterina Petrone

**Affiliations:** ^1^Grand Valley State University, Allendale, MI, United States; ^2^Leibniz-Zentrum Allgemeine Sprachwissenschaft, Berlin, Germany; ^3^Department of Linguistics, University of Michigan, Ann Arbor, MI, United States; ^4^Haskins Laboratories, New Haven, CT, United States; ^5^CNRS, Aix Marseille Univ, LPL, Aix-en-Provence, France

**Keywords:** speech planning, incrementality, crosslinguistic, individual difference, working memory, speed of processing

## Abstract

Although previous research has shown that there exist individual and cross-linguistic differences in planning strategies during language production, little is known about how such individual differences might vary depending on which language a speaker is planning. The present series of studies examines individual differences in planning strategies exhibited by speakers of American English, French, and German. Participants were asked to describe images on a computer monitor while their eye movements were monitored. In addition, we measured participants' working memory capacity and speed of processing. The results indicate that in the present study, English and German were planned less incrementally (further in advance) prior to speech onset compared to French, which was planned more incrementally (not as far in advance). Crucially, speed of processing predicted the scope of planning for French speakers, but not for English or German speakers. These results suggest that the different planning strategies that are invoked by syntactic choices available in different languages are associated with the tendency for speakers to rely on different cognitive support systems as they plan sentences.

## Introduction

Imagine three people, one French, one American, and one German, who all enter a trilingual bakery at the same moment. In unison, they all approach different clerks, and scan for a cake to buy. They all decide on a vanilla cake in the center of the display. With only one such cake on display, it becomes a sort of competition to efficiently and clearly convey the message to each clerk. In such a scenario, who is the most likely to engage in this efficient message delivery? For example, do word order differences between the languages offer an advantage to the French speaker compared to the English and German speakers? In French, the modifier *vanilla* would follow the noun *cake*: “gâteau à la vanilla.” In English or German, the modifier could precede it, as in “vanilla cake,” or it could follow it, as in “the cake with the vanilla frosting.” It is possible that this reliable post-nominal modifier placement confers an overall advantage to begin speaking more quickly for the French speaker. Another possibility, however, is that the individual cognitive differences among the speakers is a more important factor in conveying the request. For example, given the importance of working memory to ordinary cognitive tasks, will the speaker with the highest working memory span have the biggest advantage? Or given that the speed of the response is important in the scenario, is it the speaker with the fastest cognitive processing speed? There is also another possibility: What if the ideal speech planning strategy to make this request in French requires different cognitive mechanisms than the ideal planning strategies in German and English? In that case, the advantage any one speaker has depends on some interaction between the language they speak and their individual cognitive capacities. The present paper will explore that final possibility.

The cognitive process of planning sentences has, like other cognitive processes, been considered as a universal mechanism that applies to all individuals, in all situations. One manifestation of this in the psycholinguistic literature has been the search for “units” of sentence planning at the various levels of language production such as the message level, syntactic level, and phonological level. The implication of a unit of planning is that if the unit can be established, so too could one establish universal building blocks of the cognitive process itself. However, subsequent research in psycholinguistics has focused less on finding universal similarities, and more on the manner in which sentence planning processes are flexible in response to various external circumstances, linguistic circumstances, and individual differences among speakers. Such research can elucidate more than just the narrow similarities at the core of the process itself, but the full spectrum of nature of the architecture of the language production system and its relationship to other aspects of cognition. The goal of this study is to investigate interrelated effects of individual and language differences in speech planning processes. Specifically, using acoustic and eye tracking data, we examine the manner in which working memory and speed of processing might underlie the scope and characteristics of speech planning in three different languages: American English, German, and French.

### Individual Differences in Planning Scope

Speech planning is an incremental process (Kempen and Hoenkamp, 1987; Levelt, 1989), meaning that speakers do not plan a whole utterance before speech onset, but rather start speaking when a part of the utterance is ready, and continue planning as they are articulating. Research on speech planning has, over the years, tried to uncover the minimal unit of planning which is also assumed to be the characteristic unit of planning. For example, it has been suggested that planning proceeds word-by-word (Meyer et al., 1998; Levelt and Meyer, 2000; Griffin, 2001) or that the scope of planning is a syntactic phrase (Smith and Wheeldon, 1999; Allum and Wheeldon, 2007; Martin et al., 2010; Wheeldon et al., 2013). At the phonological level of encoding, different units have been proposed as well, ranging from the phonological word (Levelt, 1989; Wheeldon and Lahiri, 1997), the syllable (Meyer et al., 2003), the segment (e.g., Kawamoto et al., 2015), or a unit as large as a prosodic phrase (Keating and Shattuck-Hufnagel, 2002; Krivokapić, 2007, 2012). The variety of units of planning that have been identified, even at the same level of processing, indicates that while these units have a role in planning, a fixed unit of speech planning might not exist (Levelt, 1989; Konopka, 2012; Wheeldon et al., 2013; Kawamoto et al., 2015).

Incrementality in production has for a long time, more or less implicitly, been taken to apply automatically and universally, across speakers and across languages; however, it has been acknowledged that the scope of planning might be somewhat flexible, depending for example on the need for fluent production (Levelt, 1989), or on whether the task allows for more or less utterance planning (Griffin and Bock, 2000). Recently, various extralinguistic factors that affect the size of the planning unit have been identified, such as time pressure to complete a task, cognitive load, working memory, familiarity with sentence structure (Ferreira and Swets, 2002; Oppermann et al., 2010; Wagner et al., 2010; Slevc, 2011; Konopka, 2012; Krivokapić, 2012; Swets et al., 2014; Klaus et al., 2017). A number of studies also point to large individual variability in speech planning processes (Mortensen et al., 2008; Wagner et al., 2010; Fuchs et al., 2013; Lange and Laganaro, 2014). Such studies argue for “strategic,” rather than automatic or “architectural” incrementality, meaning that speakers can plan utterances more or less incrementally, rather than having to always use a minimal unit that triggers the next level of processing (Ferreira and Swets, 2002).

One of the cognitive factors that has been suggested to play a role in speech production is working memory (e.g., Hartsuiker and Barkhuysen, 2006; Kellogg et al., 2007; Wagner et al., 2010; Slevc, 2011; Klaus et al., 2017; Klaus and Schriefers, 2018; Ivanova and Ferreira, 2019), and a few studies to date have specifically examined the effect of working memory on the scope of planning.

**Figure 1:**
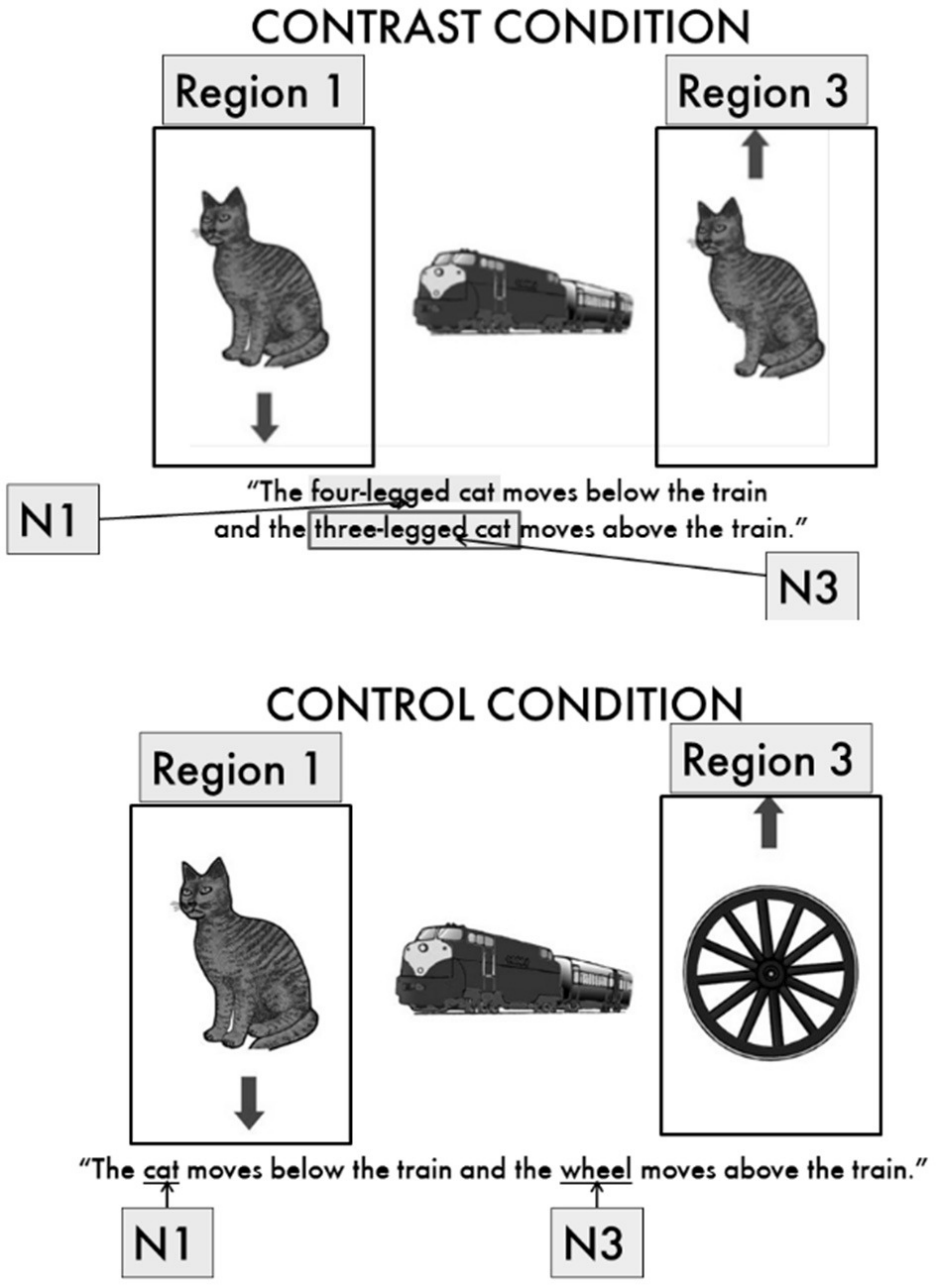
Contrast and control conditions in Swets et al. (2014). Figures 1 and 2 are reprinted from Swets (2015). With permission from Peter Lang International Academic Publishers, Frankfurt am Main, Berlin, Bern, Bruxelles, New York, Oxford, Wien, copyright 2015.

Because we used the same basic methodology in the present paper, we focus this review of the WM literature mostly on a study by Swets et al. (2014) that investigated whether individual differences in WM predict the scope of sentence planning in English. In that study, participants performed a task in which “directors” were giving commands in English based on a picture on one computer (see Figure 1) while “matchers” were manipulating objects on another screen based on these commands. During critical trials, participants uttered sentences such as “The four-legged cat moves below the train and the three-legged cat moves above the train” (see Figure 1, top image), where two objects contrasted and early disambiguation between the two objects was necessary in order for the matchers to be able to manipulate the objects. Directors disambiguated these utterances by modifying nouns, such as calling the first cat “the four-legged cat” rather than simply “the cat.” These “contrast” sentences were compared to control sentences in which no disambiguation was necessary (for example, with only one cat in the picture to be described, as in “The cat moves below the train and the wheel moves above the train,” as depicted at the bottom of Figure 1). The study used several measures to determine the scope of speech planning, including eye movement data, sentence initiation times, and whether the first noun phrase (N1 in the figure) was modified to disambiguate it from the third noun phrase (N3 in the figure) in the contrast condition.

One of the principle findings of the study came from the examination of eye movement patterns as participants planned their utterances, and how those patterns were predicted by WM scores, as measured by a variant of the reading span task (Daneman and Carpenter, 1980). The way the researchers did this was to first measure the time participants took to initiate speech by hand-coding those times. Then, those initiation times were used to calculate a measure of how far ahead speakers planned, as indexed by eye movement data. Specifically, the researchers identified the proportion of the initiation time (time before articulation began) that participants spent gazing at the third object in the display (Region 3). The rationale was that higher proportions of initiation time gazing at Region 3 were indicative of more careful advance planning—that if speakers were gathering that information prior to speech onset, they would be better able to produce an unambiguous description to the matcher. The main result from examining eye movements was that individual differences in WM predicted this tendency to gaze longer at Region 3 during the initiation time period, prior to speech onset. Specifically, speakers with high WM were more likely to gaze at Region 3 prior to speech onset than speakers with lower WM. Speakers with high WM were also more likely to modify the first noun phrase than low WM speakers. Interestingly, initiation times for contrast sentences did not vary based on WM. As Swets et al. (2014) argue, these results indicate that speakers with high working memory planned further ahead, and they did so in the same initiation time, indicating that they plan more efficiently.

Other research using individual differences approaches has also supported this link between WM and the scope of sentence planning. Petrone et al. (2011) examined the link between WM and scope of planning in German by examining fundamental frequency (F0, which is the acoustical signal corresponding to pitch in perception). The height of F0 has been suggested to be indicative of planning since there is evidence that phrase initial F0 is higher for longer than for shorter phrases [see overview in Fuchs et al. (2013)]. Petrone et al. (2011) found that phrase-initial F0 was higher for high WM speakers than for low WM speakers, indicating a longer scope of planning for speakers with high WM. Other recent studies have shown that speakers with lower WM (Bishop, 2020; Bishop and Intlekofer, 2020) and patients from medical populations associated with lower WM capacity (De Looze et al., 2018) produced shorter prosodic phrases. The researchers suggested that the reason for this could be that these speakers planned shorter chunks of speech.

Finally, Klaus et al. (2017) investigated the effects of visuospatial and verbal working memory load on the scope of planning at the phonological level and found that the scope of planning is reduced when a verbal working memory task is performed concurrently, but not when a visuospatial task is performed concurrently, thus showing the effect of (task specific) WM resources on speech planning.

These studies each provided evidence that the scope of planning is linked to WM. However, as Swets et al. (2014) pointed out, their study did not include measures of speed of processing (Salthouse, 1994) in addition to WM. For that reason, it is possible that the differences in scope of planning between high and low WM might be partially or fully attributable to speed of processing. Working memory is a set of systems that allows for short-term storage of materials necessary to execute complex tasks (Baddeley, 2010), while speed of processing refers to a general mechanism that determines speed of performance (Salthouse, 1994). Both of these capacities could play a role in the process of speech planning, as speakers need to package together chunks of utterance information, store them until they are articulated, and do all of this rather quickly. Thus, speakers could vary in their capacity for storage (buffering in WM) and/or in the efficiency of the planning process [which could be due to speed of processing differences; see discussion in Swets et al. (2014)]. This is shown in Swets et al. (2014) where, in the contrast condition (e.g., two cats in the display), speakers with high WM planned larger chunks of speech, and they did so in the same amount of time as speakers with low WM planned their (shorter) chunks, indicating that speakers with high WM were more efficient. Furthermore, high WM speakers were faster than low WM speakers in the control trials, again indicating that they were more efficient—and thus might possess faster processing—than low WM speakers.

It is also known that working memory and speed of processing are related; as summarized in Salthouse (1994) concerning age differences in working memory performance that can be related to differences in speed of processing. Thus, the WM effects observed in the two studies might be reducible to speed of processing effects. In addition, it has been reported that the prosodic planning scope of French patients with Multiple Sclerosis was predicted by measures of processing speed but not by working memory (De Looze et al., 2017), which demonstrates that aspects of speech planning do seem to rely upon speed of processing. For these reasons, it is important to establish whether the scope of speech planning can be predicted by WM over and above the variability explained by speed of processing, and vice versa.

### Cross-Linguistic Differences in Planning Scope

In addition to individual variability in the extent to which speakers might plan content in advance of articulation, there is evidence that planning scope can vary across languages as well (Christianson and Ferreira, 2005; Brown-Schmidt and Konopka, 2008; Myachykov et al., 2013; Norcliffe et al., 2015). Indeed, previous research has considered cross-linguistic factors that account for differences in language production [e.g., Montag et al., 2017; see Jaeger and Norcliffe (2009) for a review], but this work has not specifically addressed whether planning scope might vary by language. Brown-Schmidt and Konopka (2008) examined the different planning strategies employed by speakers of English and Spanish as they produced noun phrases with scalar modifiers. The experimental task required speakers to visually inspect arrays of objects as their eye movements were monitored, and to identify particular objects within the arrays to an addressee. In some cases, the object to be described was different from another object in the array only in size. In such cases, speakers were supposed to identify in their description which of the two objects was being described. For example, if an array contained two butterflies, the proper description of the object would be *the small butterfly*. Importantly, English and Spanish differ in how the two languages place the order of the noun and modifier with such scalar adjectives. Whereas, the size adjective in English is placed pre-nominally (before the noun), in Spanish, it is placed post-nominally (after the noun), as in *la mariposa pequeña (butterfly small)*. Crucially, the eye movement record indicated that English speakers fixated and planned the full noun phrase including the adjective at an earlier point in time than Spanish speakers, who were able to plan in a more “lexically incremental” (word-by-word) fashion. Hence, the scope of planning such chunks can vary by the word-order demands of a particular language.

In a comparison of planning strategies employed by Russian and English speakers, Myachykov et al. (2013) observed that speakers of the language with more flexibility in syntactic options, Russian, took longer to initiate their speech and showed larger eye-voice spans. This indicates that if languages differ in the syntactic options available to speakers as they begin to plan their utterances, these different options might draw out different planning strategies, and different cognitive resources to support those strategies as well.

Specifically, if one language's properties encourage a particular message to be encoded further in advance than another language, it is possible that such a planning strategy (larger chunks planned at a time) would be more likely to engage WM resources in the creation of those larger utterance plans [as in Swets et al. (2014)]. However, if a language encourages a more incremental strategy to encode that same message (smaller chunks planned at a time), WM may not be engaged as much—rather, perhaps cognitive processing speed might better support speakers when using this more incremental strategy. The central aim of the present study is to test that possibility by investigating the extent to which WM and speed of processing separately predict the scope of planning for similar utterances in three different languages: English, German, and French.

English, German and French are all Indo-European languages with many similarities, but there are several differences among them that may result in French being planned in a more incremental manner than English and German.

For the purposes of the present study, the main important difference among English, French in German is quite similar to the cross-linguistic differences exploited in Brown-Schmidt and Konopka (2008) and Myachykov et al. (2013). That is, when producing a noun phrase with a modifier, English and German flexibly allow modifiers to either precede or follow the noun (e.g., it would be perfectly acceptable in English to use either the post-nominally-modified *The cat with four legs* or the pre-nominally-modified *The four-legged cat*). French, on the other hand, like Spanish, does not afford speakers such flexibility. Rather, the modifier is nearly always post-nominal. Only a very small subset of adjectives can be in prenominal position, with shifts in position sometimes leading to different meanings: “la pauvre femme” (the unlucky woman) vs. “la femme pauvre” (the indigent woman). Given that previous results (Brown-Schmidt and Konopka, 2008; Myachykov et al., 2013) showed either a smaller scope of planning or a faster speech onset time in a language that forced post-nominal modifications, it is possible that French utterances in the current task will be planned in a manner that is more incremental than English and German, and therefore be more likely to tax different cognitive support systems. The question, then, is how to determine whether one language is being planned more incrementally than the others, so that the individual differences effects across languages can be interpreted with respect to the planning strategies employed in the different languages.

### Indicators of Planning Scope

The present study will focus on three types of measures that are intended to reveal something about the scope of planning: initiation time to begin speaking, pauses during speech, and eye movements before and during speech. Although initiation time is often used as an indicator of speech planning scope (e.g., Ferreira and Swets, 2002, in which speakers initiated speech more quickly to speak aloud the answers to easy arithmetic problems compared to difficult ones), its use for this purpose can be somewhat fraught as a measure by itself (Swets et al., 2013, 2014; Ivanova and Ferreira, 2019). Namely, initiation time is often not sensitive to clear differences in planning strategies that other measures reveal. For example, Swets et al. (2013) measured initiation times to begin speaking as participants described tangram objects either to a co-present addressee or without a co-present addressee. Although participants in the presence of an addressee produced more detailed descriptions, initiation time was the same in both conditions. In other words, it appeared that more planning had been carried out by the speakers with co-present addressees, but in the same amount of time taken by the speakers with addressees. Similarly, in Swets et al. (2014), participants with higher WM planned more in advance than participants with lower WM, but these effects were not revealed in initiation time measures. However, initiation time is an important starting point. If one language in the present study reveals much faster average initiation time than other languages, or if different cognitive predictors seem to operate on this variable across languages, we might assume that this language is planned in a more incremental manner in general than those other languages.

Another important measure of general planning strategies to pair with initiation time in the present study is pausing. A large body of research has identified a relationship between linguistic structure and pausing, such that speakers pause more often and longer at major syntactic boundaries, and before longer and more complex prosodic and syntactic phrases (e.g., Cooper and Paccia-Cooper, 1980; Gee and Grosjean, 1983; Ferreira, 1991; Strangert, 1991, 1997; Krivokapić, 2012; Fuchs et al., 2013; Redford, 2013), indicating that speakers use pause time to plan upcoming material.

While pause duration is often used to examine speech planning, we posit that it is useful as an indicator of local planning, but not as useful as a measure of global, incremental vs. non-incremental planning strategies. Rather, if speech planning is more or less incremental, it would seem that the frequency of pauses throughout the rest of the utterance, once speech has begun, might better reflect the overall planning strategy employed by a speaker. For example, Ferreira (1991) finds that participants added a pause within a sentence (preceding a verb phrase) when the second part of the sentence (the verb phrase) was syntactically more complex. Ferreira (1991) argues that the pause insertion occurs because speakers need additional time to plan the upcoming verb phrase. Thus, an increase in the number of pauses in an utterance indicates that speakers did not plan as far in advance of articulation, thus requiring additional planning time during articulation. Fewer pauses would reflect a greater degree of advance planning scope. For these reasons, we regard it to be useful to add pause frequency as another potential indicator of general planning strategy differences among languages. A further finding is that there is large individual variability in pause occurrence and duration (e.g., Goldman Eisler, 1968; Cummins, 2002; Fuchs et al., 2013). These individual and cross-linguistic differences have not been examined in relation to speech planning processes.

Although both initiation time and pause occurrences are useful measures to portray global planning tendencies, the measure that should be most indicative of local planning scope, and sensitive to the differences in planning strategies among languages and individuals, centers on the same eye movement patterns that were examined in the Swets et al. (2014) study. In Swets et al. (2014), it was the measure of how long participants gazed at the final region to be described in the display prior to speech onset that offered the best view of how planning scope can vary among individuals with different WM span.

### Research Questions

The present study uses the same methodology as the Swets et al. (2014) study described above, but performed in a cross-linguistic manner, with the additions of speed of processing as an individual differences measure and pause frequency as an additional measure of speech planning tendencies. As in that study, we are investigating the scope of planning at the interface between the level of message planning and the level of utterance planning. There are two main questions that this study seeks to address. One is whether we find evidence for differences in planning strategies, or scope of planning, when planning similar utterances in three different languages—American English, German, and French—within a specific, controlled speaking task. A second question is whether effects of individual differences in working memory [as found in Swets et al. (2014) and Petrone et al. (2011)] and speed of processing [as in De Looze et al. (2017)] on the scope of planning vary among these different languages; and whether such effects are independent of each other. Then, to put these two questions together, we investigate whether any observed cross-linguistic differences in planning strategies seem to recruit different cognitive mechanisms.

We aim to distinguish between two broad theoretical accounts. Both accounts come from the perspective that the scope of planning in language production is, in fact, flexible and adaptive to different circumstances such as time pressure and WM, as has been suggested in previous research (e.g., Swets et al., 2014). But where they differ is in how that flexibility manifests across different languages. On one view, this flexibility is a consistent architectural feature of human speech planning that is similar across languages. Let us call this the Partly Dynamic view. This view would assume that speech planning relies on the same cognitive support systems to the same degree in different languages. So, for example, it is possible that English, German, and French speakers will show similar planning strategies and scope of planning, and that the relationship between WM/speed of processing and scope of planning will be equivalent to these other languages. In that case, we may observe that not only do speakers of all three languages plan these utterances with roughly the same scope in general, with similar initiation times and pause rates, but that WM and speed of processing predict planning scope in the same manner for speakers of all three languages. Under this scenario, the cross-linguistic differences may be smaller than the individual differences that may exist within each language sample, and a speaker of one language won't benefit differently from higher WM or processing speed than speakers of another language.

On a Fully Dynamic view, however, cognitive mechanisms may be recruited differently to support planning within the different languages. On this view, if a language's grammatical properties invite a more incremental planning strategy, different cognitive resources play a larger role in planning compared to a language with different properties. For the present study, this view would predict that French speakers will tend to plan in a more incremental manner than speakers of the other two languages with flexible modification, because they can trust that they can modify the first noun on the fly, post-nominally. On this view, French speakers, adopting a more incremental planning strategy, would show shorter initiation times than English and German speakers, as well as higher pause frequency. Then, if these different planning strategies recruit different cognitive resources, we might observe that for languages that are planned in a more incremental manner, it is processing speed that shows a relationship to various measures of speech planning, whereas for languages that are planned in a less incremental manner, it is working memory that supports greater degrees of advance planning. To again return to the trilingual bakery, it may be that the French speaker has an advantage in making the cake request sooner, and that this advantage becomes even greater in the presence of higher processing speed; whereas the English and German speakers may be starting their request later, but with fewer pauses, and are helped more by additional working memory.

## Method

The investigation involves three studies using a similar experimental design. Study 1 will focus on American English speakers, Study 2 on German speakers and Study 3 on French speakers. The principal difference in methodology between Studies 1 and 2 was the model of eye tracker that was used to collect the eye movement data, but data from each eye tracker is compatible with data from each other eye tracker, and was analyzed in the same way after taking different sampling rates into account. There were no methodological differences between Studies 1 and 3.We note that the three studies were conducted each in a different laboratory—in the United States, Germany, and France.

### Participants

Participants for Study 1 were 30 native speakers of English from Grand Valley State University who received course credit for their participation. Participants for Study 2 were 31 native speakers of German, residents of Berlin that were recruited from a participant database. Each participant was paid 10 Euro for their participation. Study 3 was carried out with 32 French native participants from the Aix-of Provence area. They received 10 Euro for compensation. All participants had a similar educational background and were students at the university.

### Design

The study used a design with one within-subjects manipulation, Display Type, which was whether an array of three objects being described by the Director contained an object that required a verbal contrast with another object in the same array (contrast condition) or contained three different object types (control condition). There were also two continuous predictors in the study: the working memory and processing speed measures. In the analyses below, we combine data from the three studies to investigate language spoken as a predictor variable.

### Apparatus

We collected eye movement data using the table-mounted EyeLink 1,000 eye tracking system for Study 1 (American English) and Study 3 (French speakers). The system sampled eye position data once every 2 ms and was facilitated by Experiment Builder software. For Study 2 (German speakers) eye movement data were recorded using the head-mounted EyeLink I eye tracking system, which sampled eye position data once every 4 ms. The calculations that we made for eye gaze measurements were adjusted based on the sampling rate differences. We used Experiment Builder software to facilitate the production task.

### Materials and Procedure

All instructions and material text were translated from English into German (Study 2) or French (Study 3) by native speakers of the respective language (for Study 2 by the second author). The procedure of Study 1 was repeated for Study 2, with the experimenter serving the dual role as Experimenter/Matcher for each participant in Study 2. To help ensure a consistent procedure for each site, the author who established the procedure for Study 1 in English was present for the first set of participants who were run in both Study 2 (German) and Study 3 (French).

Participants acted as Directors in this experiment. Directors sat in front of a computer monitor and an Eyelink camera which the experimenter then calibrated. A headset microphone recorded each Director's utterances. Directors read a set of written instructions presented on the screen, then engaged in five practice trials to learn how to produce target utterances when presented with arrays such as in Figure 1. The instructions asked participants to interpret the arrows near the objects as the directions in which they were supposed to move relative to the center object. For example, in Figure 1, the downward-pointing arrow below the left cat should be described as “(the cat) moves below the train,” while the upward-pointing arrow above the right cat should be described as “(the cat) moves above the train.” We asked participants to combine those two movement commands into one sentence, with sufficient detail for a Matcher to follow them as object-movement commands. For example, in response to Figure 1, the target utterance could be “The four-legged cat moves below the train, and the three-legged cat moves above the train.” If an arrow pointed toward the center object, they were to say that the object “moves next to” that center object.

Participants practiced the movement commands by seeing five displays using monosyllabic real world objects that were not present in the experimental displays. This practice session gave participants the opportunity to learn how to produce a target utterance in the desired format, including the movements indicated by different arrows (above, below, and next to).

**Figure 2:**
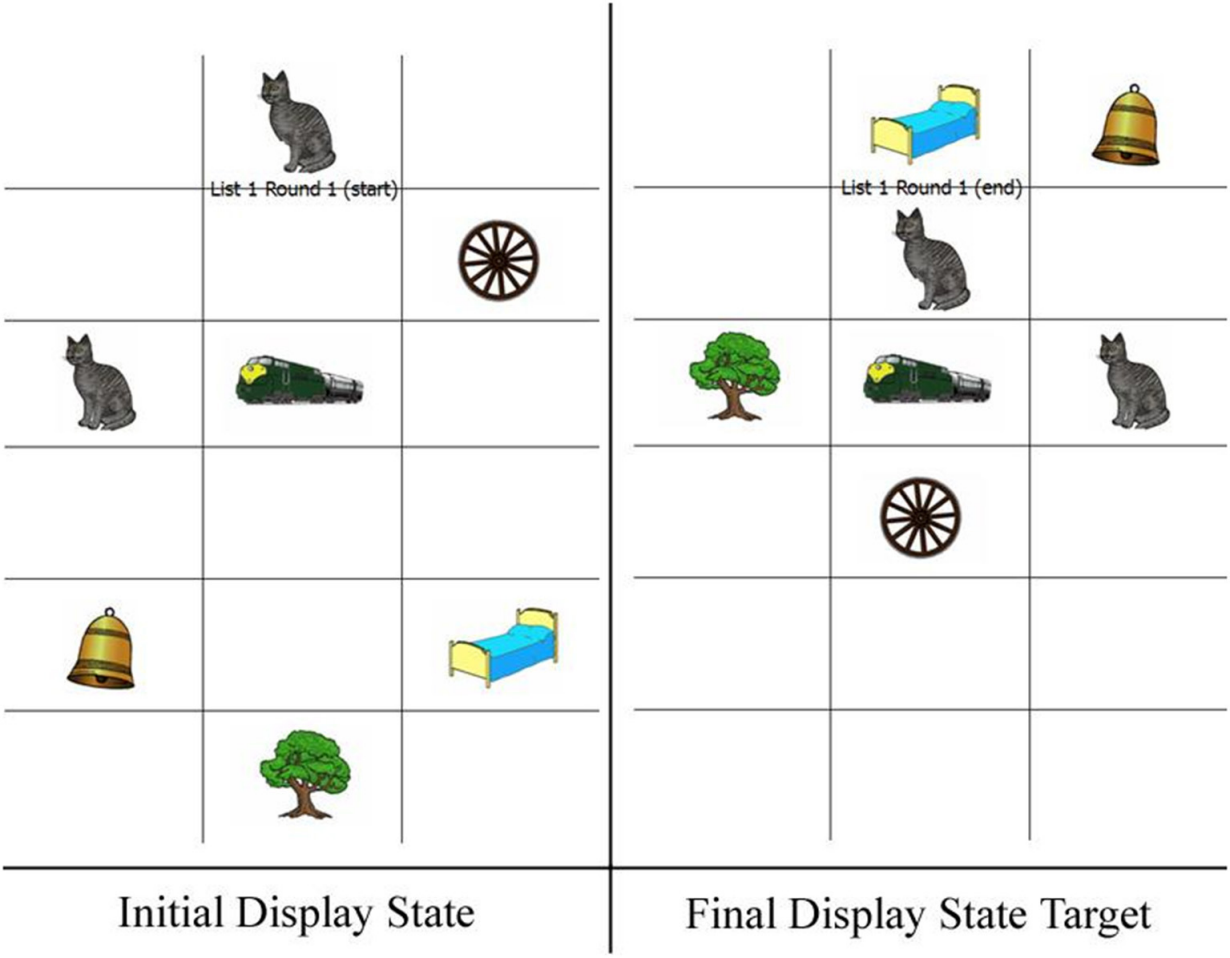
Examples of Matcher displays. The left side shows the initial state of a display before the beginning of a given round. The right side shows target state of the display after all commands had been given for a round.

The instructions further explained that a Matcher would be using the Directors' descriptions as instructions for where to move objects on their own Matcher boards (see Figure 2). Although Directors did not see the Matcher boards as they provided their descriptions/commands to the Matcher during the actual experiment, they were shown an example of the Matcher boards during these practice trials. Seeing the Matcher boards during the practice trials helped Directors to understand better why they could not simply say something such as “The left cat moves above the train” in beginning to describe Figure 1: The Matcher boards looked different than what the Directors saw, so it was important to provide enough detail about the objects to the Matcher to accurately move the pieces around their board without relying on directional cues such as “left” and “right.”

To begin each trial, Directors were required to stare at a fixation cross on the left side of the screen and press the space bar. After that, one of the three-object arrays (such as in Figure 1) would appear on the screen, which the Director would then describe. The experimenter served as the Matcher. After each utterance by the Director, the experimenter used a laptop to move objects around their image grids, and asked for clarification if the Director provided insufficient detail during the initial description. For example, if the Matcher heard “The four-legged cat moves below the train,” he or she would move the four-legged cat into a slot on their Powerpoint grid directly below the train's slot. Once the experimenter made the corresponding object movements, he or she said “OK” to indicate to the Director that it was time to move on to the next trial. The experimenter sat at a different table than the Director, where it was possible to both monitor the calibration accuracy of the eye tracker while also participating in the matching process. Although it was possible for the Director to turn his or her head to see the Matcher/experimenter, eye contact during the experiment was rare.

#### Matcher Materials

The materials for the Matcher consisted of 8 grids, one for each of the 8 rounds of the matching game. Grids contained 24 squares, with 3 columns and 8 rows of squares in each grid (see Figure 2 for an example). Each grid contained 6–8 objects, depending on how many objects would be mentioned by the Director during each round. These image grids allowed for the movement of each individual image from one slot to another by clicking and dragging it to an empty slot using a mouse. The matching game that began each session consisted of 8 rounds, and each round included 2 experimental utterances and 6 filler utterances.

#### Director Materials

The 48 individual objects used as stimuli were the same as those used in the Swets et al. (2014) study, which were adopted from a database of color images that can be found at the website https://wiki.cnbc.cmu.edu/Objects (Rossion and Pourtois, 2004). These 48 objects were combined into larger arrays of three objects apiece and used as the stimuli to elicit the Directors' target utterances (e.g., “The cat moves below the train and the wheel moves above the train”). All objects were real-world objects with high naming accuracy and low reaction times (Rossion and Pourtois, 2004). The 48 individual objects were combined in various 3-object permutations to create 16 experimental arrays, with each of the three objects aligned horizontally across the screen. Each of the 16 arrays was created with both a control version and an experimental, contrast version, with 2 target and 6 filler arrays presented per matching round. Whereas control displays had three unique objects, in contrast displays, the right-most unique object from control displays was replaced with a slightly altered token of the left-most object. Because the right-most object (in Region 3) during Contrast trials was always the slightly altered token, it was also the generally the more salient of the two. Control and contrast displays were counterbalanced across two lists, and each participant only saw one list. Figure 1 shows an example of a contrast display. In each array, arrows indicated the directions the objects were supposed to move. Half of the experimental arrays depicted the first object was moving up and the third object moving down, and the other half depicted the opposite. A total of 96 fillers distributed across the two lists included directions such as *next to*, and occasionally involved only one moving object, in order to diversify the types of utterances Directors produced during the experiment. To ensure that the Director had not already created a description of an object prior to appearing in an experimental trial, filler items containing objects of interest did not appear until after such experimental trials had already been executed.

### Working Memory and Speed of Processing Tests

After completing the matching game, the experimenter gave participants a paper packet that included WM and processing speed tasks. Materials for the WM assessment were adopted from a variant of the reading span task in which participants completed 8 sets of trials totalling 36 items. There were two sets with 3 items, as well as two sets with 4, 5, and 6 items. The 36 items were derived from the items originally used in Daneman and Carpenter (1980) and subsequently adapted based on Turner and Engle (1989). Items were presented in Powerpoint on a laptop. For each item, the first presentation screen showed a sentence with a word printed underneath. The sentence was either semantically plausible (e.g., “The woman planted flowers on her patio”), or implausible (e.g., “After dinner the couple had a glass of tree”). To elicit yes/no semantic plausibility judgments, a question mark appeared after each sentence.

To begin the working memory task, participants viewed a set of written instructions, followed by a practice session consisting of two-trial set. For each set, including the practice trials, the answer packet had two pages. On the first page, there were six lines that consisted of YES and NO. If the sentence on the laptop screen made sense, they were to answer YES, and they were to answer NO if it did not make sense (participants only filled out all six items on trials with a set-size of six). On the second page for each set, there appeared six numbered, blank lines where participants were to write down the words that had appeared under the sentences that they were judging. The Powerpoint file presented each sentence/word item for 5 s, and after the last item in a set, a prompt (three question marks) appeared on the screen to indicate that it was time to turn the page from the YES/NO judgments to the page on which participants wrote down the memory-task words.

After the last working memory task item, participants completed the assessment on speed of processing. To measure this, we used the letter comparison task from Salthouse (1996), in which participants made a series of judgments regarding the similarity of sets of letters. We chose letter comparison as a measure of processing speed because it has been shown to tap into the specifically cognitive components of speed rather than motor components (Redick et al., 2012), and because like reading span, it is a task that uses language-oriented stimuli (letters) rather than numbers or spatial patterns. First, an instruction page oriented participants to the task. The instructions asked participants to “determine whether two strings of letters are the same or different. If the letters in the two strings are the SAME, write an S on the line between them. If they are DIFFERENT, write a D on the line. Please try to work as rapidly as you can, writing an answer for each comparison.” After 3 practice items, the experimenter told the participant to turn the page to the first set of letter comparisons, and gave the participant 30 s to work on the first page. After 30 s, the experimenter then timed a second page of letter comparisons, once again for 30 s.

### Data Processing and Analyses

#### Annotation of Acoustic Data

To obtain the measures concerning noun phrase modification, initiation time, and pause frequency, experimenters trained research assistants to perform manual speech annotations in Praat. Each target utterance was considered under the following framework of a target utterance as seen below.

[Initiation Time] The N1 moves below the N2 and the N3 moves above the N2.

We coded the first noun in each utterance, N1, as being un-modified (e.g., “the cat”), modified pre-nominally (e.g., “the four-legged cat”) or modified post-nominally (e.g., “the cat with four legs”). There were three different measures produced by this coding process. First, we coded whether a speaker produced a fluent modification of N1 (1 = fluent modification, 0 = no modification or late modification). A fluent modification would not include instances of late modification, when speakers first articulated a bare noun (e.g., “the cat”), continued on with the sentence (e.g., “moves below the”), then realized that there was a second cat in the display, causing them to go back and produce a modification of N1 after having moved on from that utterance section. We assume that if a speaker has fluently modified N1, they have realized early on that there is a need to distinguish N1 from N3. We also coded separately whether each N1 was modified pre-nominally (1 = fluent pre-nominal modification, 0 = no fluent pre-nominal modification), and whether each N1 was modified post-nominally (1 = fluent post-nominal modification, 0 = no fluent post-nominal modification).

We calculated Initiation Time as the duration from the onset of stimulus presentation on the screen to the onset of the first word of the speaker's description (*The)*. Initiation times were log-transformed for statistical analyses.

Pauses were annotated by hand in Praat (Boersma and Weenink, 2018). Because Directors all completed the task in their L1, and were therefore likely to be quite fluent, we considered any silence after the onset of speech longer than 100 ms as a pause [for discussion see Hieke et al. (1983)]. The frequency of pauses was automatically calculated after they were entered in by hand.

#### Eye Tracking Data

Once initiation times were annotated in Praat, those time windows could be used to integrate the temporal data from the speech onset times with eye movement data collected from the eye trackers. Each display was divided in three equal-sized regions, with Region 1 being the furthest left, and containing the object that corresponded to the first noun to be described in each target utterance (N1). Region 2 contained the center object in each display (N2). Region 3 contained the object to the right, which corresponded to the third noun to be described in each target utterance (N3). Although we calculated the proportion of initiation time that speakers were gazing in each region separately, we will focus our analyses just on the proportion of initiation time that speakers gazed at Region 3 of each display (Every trial began with participants gazing at the fixation cross that was located in Region 1, making it less interesting as a measure of lookahead than Region 3. Region 2 was unrelated to advance planning of the possible contrast between the first and third objects). To obtain this measure, we created a script in Matlab. The script calculated how many eye tracking samples showed that the speaker was looking at Region 3 (the third object of the display) during the Initiation Time window for each trial. Recall that eye tracking samples were collected every 2 ms during English and French trials and every 4 s during German trials. Based on this procedure, we were able to calculate the proportion of eye tracking samples during the initiation time window of each trial that speakers were gazing at Region 3 of the display. This was possible by leveraging the fact that samples were taken by the eye tracking system on a known millisecond time scale. In our analyses of these proportions, the total number of samples was included in the models along with the proportion to make sure that overall initiation time was part of the weighting in the models.

#### WM and Speed of Processing Tests

To obtain the WM score of a participant from the reading span task, an item was considered correct if a participant correctly judged a sentence's plausibility and also correctly recalled the word in the correct serial position. The total number of correct items (range: 0–36) in the reading span task was used as a continuous measure of working memory.

For letter comparison, we counted the number of correct answers (range: 0–21) on each of the two pages and used the average score as the continuous measure of processing speed.

#### Statistical Analyses

All statistical tests were performed in the R software environment (R Core Team, 2018, v. 3.5.1).

Linear regression models were used for comparison of neuropsychological scores across the three languages. Raw scores of working memory (as obtained from the reading span task) and speed of processing (as obtained from the letter comparison task) were entered as dependent, numerical variables. Language (English/German/French) was the only independent variable.

Furthermore, a series of mixed models was run on the first noun phrase (N1) modifications, eye movements, initiation times, and number of pauses. For N1 modifications, three generalized linear models with mixed effects were run based on binomial distribution. The first model was aimed at examining the likelihood that speakers fluently modified N1 at all (1 = fluent modification, 0 = no fluent modification), while the other two models evaluated whether, in the case of N1 modifications, N1 was modified either pre-nominally (e.g., “the four-legged cat”) or post-nominally (e.g., “the cat with four legs”). For eye movements, generalized linear models with mixed effects were run on the proportion of time speakers spent gazing in Region 3 prior to speech onset, i.e., during the utterance window defined as initiation time. Because of the high number of trials during which speakers did not gaze at Region 3 (where each time-based sample was coded 0 = not gazing at Region 3, and 1 = currently gazing at Region 3), especially in the control condition, the data for this measure had a large number of zeros present. Because zero-inflated data is known to create problems in model convergence for mixed effects models, we based our models on betabinomial distributions, which are more robust in modeling zero-inflated data. As is standard practice with response latencies and reaction times, we fit our linear mixed models to logarithmically transformed initiation times (Baayen, 2008). Poisson generalized mixed models were fit to number of pauses during the production of the whole utterances.

The generalized and linear mixed models were applied to test the effects of the factors working memory (WM), speed of processing (SP), display type (contrast/control), and language (English/German/French; English was entered as reference level) on N1 modifications, gaze times, initiation times, and number of pauses. We entered English as the reference level to which German and French were hence compared. We chose English as the reference level because that language had already been examined [in Swets et al. (2014)], and we wanted to be able to compare the new languages tested to the language with existing data patterns. In our corpus, working memory ranged from a score of 5 up to a score of 32 (e.g., there was no participant scoring the lowest or the highest possible values, i.e., 0 and 36), while speed of processing ranged from 6.5 to 17.5 (none scored the lowest or the highest possible values, i.e., 0 and 21). Three-way interactions between display type, language and individual cognitive scores (WM and SP) were also calculated. Working memory and speed of processing scores were centered based on group means by language, and these centered scores were entered as predictors for each dependent variable.

Speakers (1–93) and items (1–16) were included as random intercepts. The factor item corresponded to the 16 visual stimuli. Random slopes for display type, language, working memory, and speed of processing were included both per subject and per item. For categorical dependent variables (gaze times and number of pauses), we report the *p*-value of the generalized linear mixed models. For initiation times, *p*-values were obtained through the *LmerTest* package. The cut-off point for significance was set at *p* < 0.05.

## Results

First, we present data from the individual differences measures of WM and processing speed from the three language samples. Then, we introduce data from the speech measures. The first speech measure we report is in regards to the way speakers modified the first noun phrase—whether they did so at all, whether they did so pre-nominally, and whether they did so post-nominally. The next two measures we will present are initiation time and pause frequency, which help to establish whether the three languages may have been planned differently in the present task. Then, we examine eye movements to investigate whether the scope of planning in each language differs among each language, and whether different individual cognitive capacities might be used differently in how that scope of planning varies. Full model outputs are given in the Appendix.

### Working Memory and Speed of Processing

Working memory score was on average 17.3 for English (min = 5, max = 30), 19.6 for French (min = 11, max = 27) and 21.09 for German (min = 5, max = 32). The speed of processing score was 12.9 for English (min = 9.5, max = 17.5), 11.1 for French (min = 8, max = 15.5) and 11.4 for German (min = 6.5, max = 16). English speakers showed significantly lower working memory scores than French [*β* = 2.24, *SE* = 0.39, *t* = 5.70, *p* < 0.001], and German [*β* = 3.73, *SE* = 0.39, *t* = 9.47, *p* < 0.001] speakers. Conversely, English speakers had significantly higher scores for speed of processing than French [*β* = −1.84, *SE* = 0.14, *t* = −13.36, *p* < 0.001] and German [*β* = −1.47, *SE* = 0.14, *t* = −10.67, *p* < 0.001] speakers.

For each language, we calculated Pearson correlations between reading span and speed of processing scores. Although the two measures correlated positively among the French sample (*r* = 0.41, *p* < 0.05), they did not correlate significantly among the English or German samples. As noted in other research, it is somewhat surprising, but not uncommon, to find a lack of correlation between processing speed and WM (Redick et al., 2012).

### Noun Modifications

Table 1 presents the proportions of utterances in which speakers fluently modified the first noun phrase (N1) broken down by language (English, French, and German) and display type (contrast and control). In all three languages, as expected, speakers were more likely to modify N1 in contrast displays than control displays. There were no significant differences in total fluent modifications across the three languages. However, French N1 modifications in the contrast display condition were only produced post-nominally, whereas German and English modifications showed a rather balanced mixture of pre- and post-nominal modifications. Our statistical models confirmed that the only effects that were significant were in the measures of pre- and post-nominal modification likelihood, where French speakers were less likely than English and German speakers to modify N1 pre-nominally [*β* = −7.86, *SE* = 1.5, *z* = −5.02, *p* < 0.001], but more likely than speakers of English and German to modify post-nominally [*β* = 2.007, S*E* = 0.77, *z* = 2.5, *p* = 0.009]. No other main effects or interactions were significant for any of these measures.

**Table 1:** Proportions of utterances in which speakers modified the first noun phrase.

	**Total fluent modifications**		**Prenominal modifications**		**Post-nominal modifications**	
	**Contrast**	**Control**	**Contrast**	**Control**	**Contrast**	**Control**
	***M* (*SD*)**	***M* (*SD*)**	***M* (*SD*)**	***M* (*SD*)**	***M* (*SD*)**	***M* (*SD*)**
English	0.68 (0.47)	0.03 (0.18)	0.38 (0.49)	0.03 (0.18)	0.31 (0.46)	0 (0)
French	0.60 (0.49)	0.03 (0.16)	0.004 (0.06)	0 (0)	0.59 (0.49)	0.03 (0.16)
German	0.70 (0.46)	0 (0)	0.32 (0.47)	0 (0)	0.38 (0.49)	0 (0)

*Mean proportions are shown with standard deviations in parentheses*.

### Initiation Times

**Figure 3:**
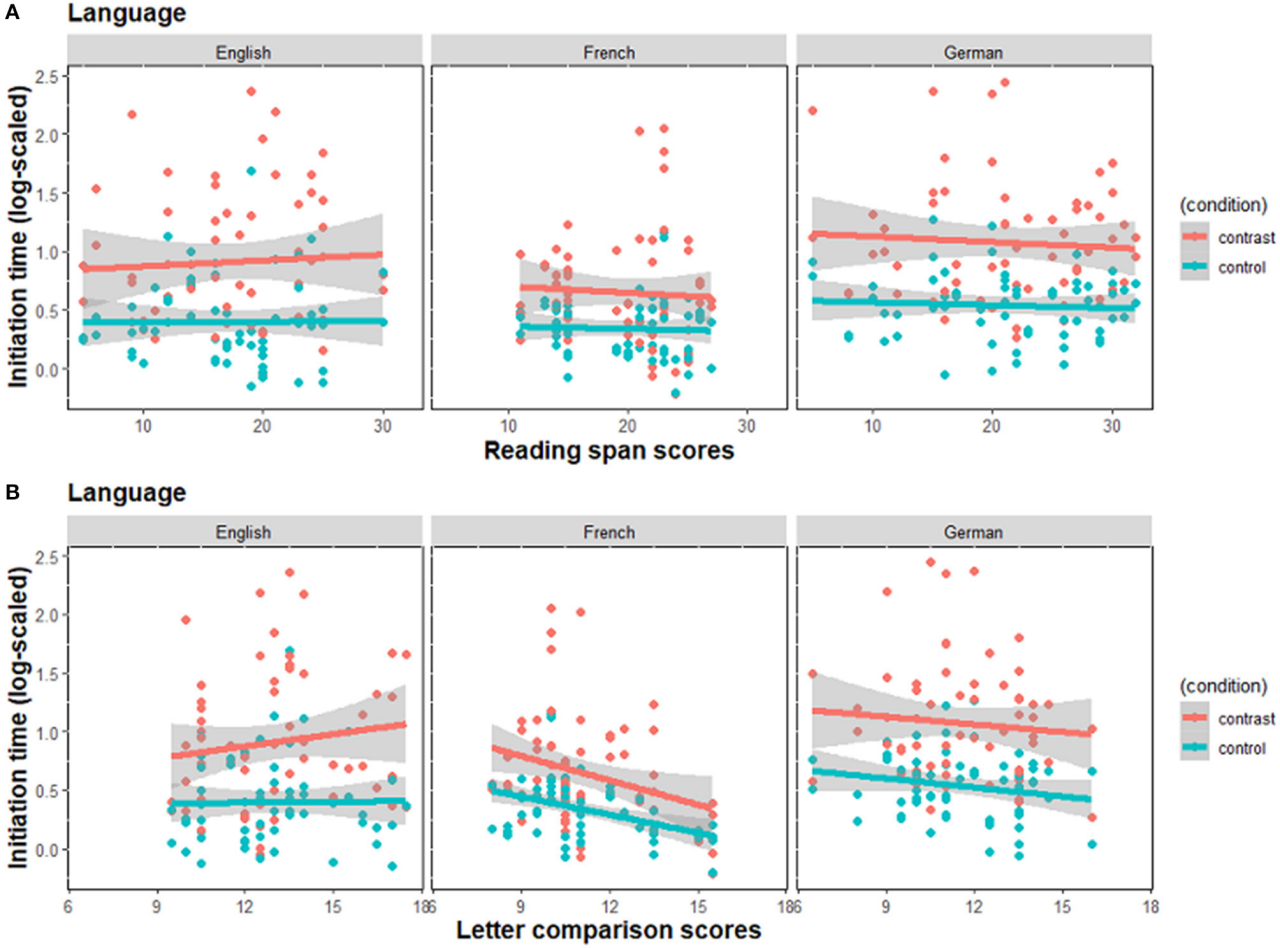
Log-scaled initiation time as a function of **(A)** working memory scores as measured by the reading span task, and **(B)** speed of processing scores as measured by the letter comparison task. The gray bands represent 95% confidence interval for the regression lines. Contrast, Contrast condition; control, Control condition.

Table 2 presents descriptive statistics for initiation times, pause frequency, and our Region 3 gaze measure, broken down by language and display type, but not by WM or speed of processing. Figure 3 shows the relationship between both working memory (top 3 plots) and processing speed (bottom 3 plots) with utterance initiation times. One clear trend visible in the figure is that for English, German and French speakers, there was a significant effect of display type (contrast vs. control) that was confirmed statistically [*β* = −0.47, *SE* = 0.079, *t* = −6.08, *p* < 0.001], such that the contrast condition had longer initiation times than the control condition. Moreover, French speakers produced speech with a shorter initiation time than English speakers [*β* = −0.23, *SE* = 0.091, *t* = −2.55, *p* = 0.011], while there was no difference between English and German speakers. English speakers showed a larger difference between the control and contrast condition in display type than the French speakers [*β* = 0.18, *SE* = 0.079, *t* = 2.28, *p* = 0.025]. Furthermore, for English and German, neither working memory nor speed of processing significantly predicted initiation time, nor did their interactions with display type.

**Table 2:** Descriptive statistics (means and standard deviations) for the three dependent variables, by language and display type (contrast vs. control).

	**Initiation time in ms**		**Number of pauses per utterance**		**Proportion of initiation time gazing at region 3**	
	**Contrast**	**Control**	**Contrast**	**Control**	**Contrast**	**Control**
	***M* (*SD*)**	***M* (*SD*)**	***M* (*SD*)**	***M* (*SD*)**	***M* (*SD*)**	***M* (*SD*)**
English	2,921 (2,927)	1,601 (1,277)	3.04 (1.95)	1.47 (1.18)	0.21 (0.16)	0.02 (0.06)
French	2,183 (1,746)	1,450 (540)	4.14 (2.92)	2.46 (1.79)	0.18 (0.16)	0.02 (0.06)
German	3,340 (2,861)	1,783 (665)	2.47 (2.11)	1.04 (1.02)	0.21 (0.15)	0.04 (0.07)

One effect that only emerged in French was a negative correlation between initiation time and speed of processing, such that, in the contrast condition, the higher the speed of processing, the shorter the initiation times [*β* = −0.10, *SE* = 0.042, *t* = −2.46, *p* = 0.015]. Hence, on average French speakers initiated speech before speakers of English, and French speakers with shorter initiation times also tended to be the same individuals who had the highest speed of processing scores. The remaining measures of planning that we report help to place this effect in a larger interpretive context.

### Number of Pauses

**Figure 4:**
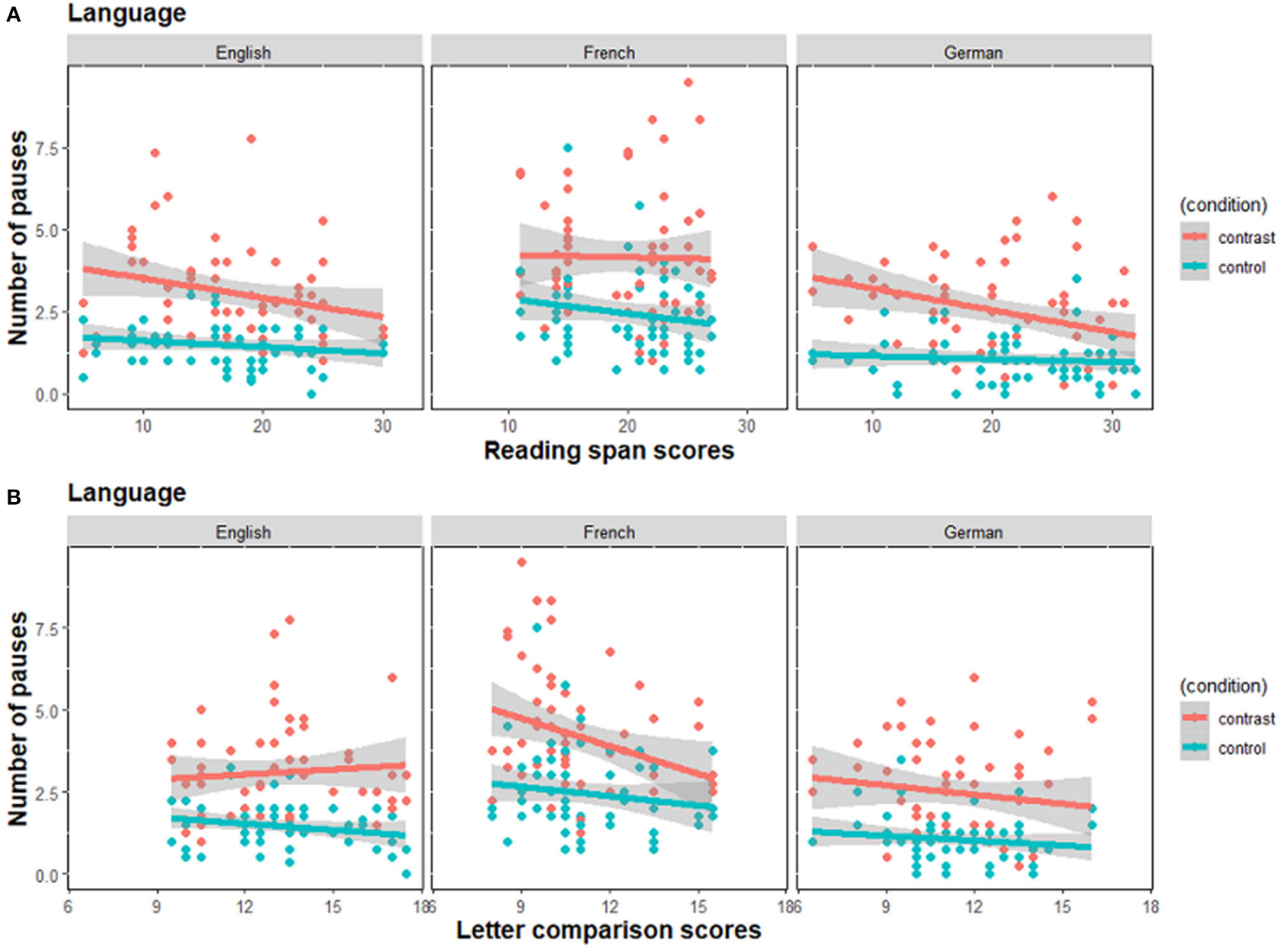
Average number of pauses per utterance as a function of **(A)** working memory scores as measured by the reading span task, and **(B)** speed of processing scores as measured by the letter comparison task. The gray bands represent 95% confidence interval for the regression lines. Contrast, Contrast condition; control, Control condition.

Figure 4 shows the relationship between pause frequency and the two individual differences measures in each language. In general, the number of pauses was higher in the contrast than in the control condition [*β* = 0.73, *SE* = 0.10, *z* = 7.31, *p* < 0.001]. Differences between English speakers and French and German speakers were also found: German speakers produced relatively fewer pauses than English speakers [*β* = −0.27, *SE* = 0.12, *z* = −2.19, *p* = 0.029] and French speakers produced more pauses than English speakers [*β* = 0.27, *SE* = 0.12, *z* = 2.25, *p* = 0.024]. The effects of the other fixed factors, predictors, and their interactions were not significant.

These results, taken along with the evidence from initiation times, suggest that the French speakers adopted a planning strategy that was more incremental in nature than English and German speakers. Specifically, French speakers engaged in more planning during articulation than speakers of the other languages in the present study, with a shorter scope of planning.

### Eye Movements

**Figure 5:**
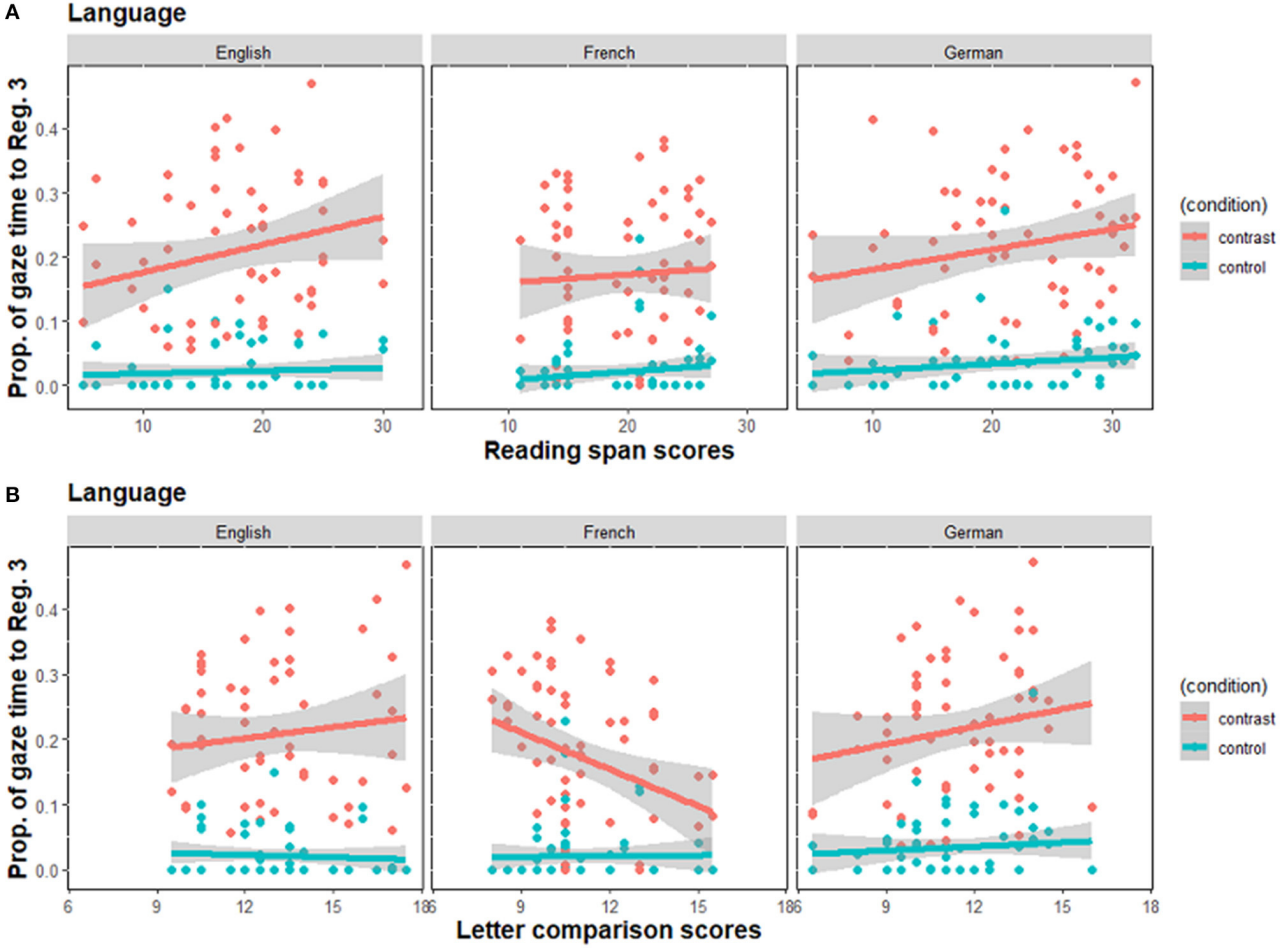
Proportion of gaze time to Region 3 per utterance as a function of **(A)** working memory scores as measured by the reading span task, and **(B)** speed of processing scores as measured by the letter comparison task. The gray bands represent 95% confidence interval for the regression lines. Contrast, Contrast condition; control, Control condition.

Figure 5 shows the relationship between the proportion of initiation time participants spent gazing at Region 3 and the two individual differences measures in each language. Inspection of the plots in this Figure suggest that once again, in English, German and French, there was a significant effect of display type: Prior to speech onset, speakers gazed less often to Region 3 in the control than in the contrast condition type [*β* = −3.44, *SE* = 0.36, *z* = −9.47, *p* < 0.001]. We did not find an interaction between display type and language. Concerning the effects of individual variation, we found only one interaction between cognitive capacities and language. In French, we found a negative relation between speed of processing and gaze time proportion, such that French speakers with higher speed of processing spent a smaller proportion of the initiation time looking ahead to Region 3 [*β* = −0.33, *SE* = 0.15, *z* = −2.13, *p* = 0.033].

Similar to the effect observed for number of pauses, we see in Figure 5 that French speakers with high speed of processing preferred a more incremental strategy of planning, as they spent less time gazing at Region 3 than French speakers with low speed of processing.

## Discussion

In examining the full set of results from measures of initiation time, pause frequency, and eye movement patterns prior to speech onset, the most notable pattern that we observed is that in the present study, the scope of planning for speakers of French, which requires speakers to modify nouns post-nominally, was predicted by different cognitive factors than for speakers of English and German. Unlike those other two languages in this sample, the French speakers showed an association between measures of planning scope and speed of processing. Not only did speed of processing, as measured by the letter comparison task, show a relationship with speech onset time, but it also predicted the extent to which French speakers would inspect Region 3 of the display prior to speech onset, a measure that indexes advance speech planning. There is also evidence that French speakers, by pausing more often than speakers of English, delayed more of the job of planning speech until after they had begun to speak. Our interpretation of this pattern of results is that French speakers in the present task adopted a more “incremental” planning strategy. Under such a strategy, speakers value speed over careful advance planning, in which case overall processing speed would be of greater benefit compared to linguistic circumstances in which speed is not the most important factor. This could help explain why French showed effects of processing speed on initiation time, whereas the less incrementally planned languages did not.

Importantly, no measures of advance sentence planning in either English or German were predicted by individual differences in WM or speed of processing. The effect found in Swets et al. (2014) that, in English, WM correlated positively with the tendency to look ahead to Region 3 prior to speech onset, did not replicate with significance in the present study (although the direction of the effect was the same—more on this below). However, if we take into account that previous study (Swets et al., 2014), the present results for English and German, and combine those with the novel processing speed data from French, we are able to draw two principle conclusions.

First, we find evidence that different cognitive resources gain or lose importance to the process of planning in advance depending on the linguistic properties of the language being planned. The evidence that supports this possibility comes primarily from the eye movement measure of the proportion of initiation time that speakers gazed at the furthest-right region in the display that they described. Whereas, this measure did not correlate with speed of processing for our English and German samples, this measure correlated negatively with speed of processing for our French sample. These results suggest that the ways in which individual differences in cognitive capacities predict the scope of sentence planning could themselves vary cross-linguistically, and are consistent with the Fully Dynamic view of the relationship between language spoken and individual differences in cognitive capabilities.

Another conclusion that we draw has to do with the separability of different cognitive factors that were tested in our statistical models. We found that the French speakers in our sample showed an association between speed of processing and advance sentence planning. The fact that we included WM as a predictor in the same model, and that speed of processing predicted advance planning scope over and above the variance due to WM, allows us to rule out the possibility that the relationship between speed of processing and planning scope can be attributed to a WM explanation. Although one of the motivations for this approach was to discover whether the effects that links WM to advance planning in English can be attributed to speed of processing, the fact that we did not find a statistically significant replication of that effect in the present study does not allow a conclusion to be drawn about that issue.

Although it may be tempting to draw broad conclusions about the cross-linguistic differences that we found in general planning strategies, we would like to emphasize that such generalizations are not afforded by our data. We do not claim that French speakers generally plan more incrementally than English speakers, nor that speed of processing will always be more important for French speakers for that reason. Rather, the data suggest that under circumstances when a language invites a particular planning strategy (more incremental in French for these particular utterances), different cognitive factors may be adaptively deployed to facilitate that particular strategy. This notion is consistent with the overall theory that planning is flexibly adaptive to the external, linguistic, and internal circumstances that all come together to facilitate efficient speech planning. Due to the complex dynamics of the interactions between those three sources, one should not expect to observe consistent, generalizable planning strategies within a certain language, nor should one expect to observe a consistent reliance on certain cognitive resources for all speakers of a certain language. In fact, we suggest exactly the opposite: The inherent flexibility and adaptability of human speech planning to linguistic, cognitive, and external factors should lead us away from creating those kinds of broad generalizations.

Let us return once more to the trilingual bakery. Although we cannot use our data to determine with any certainty whether any given speaker of any given language is likely to begin speaking sooner, or engage in the most efficient planning strategy in the attempt to order the vanilla cake, our proposal is merely that cross-linguistic and individual differences ought not be separated from each other. Based on our study, the French speaker could have an advantage in the situation by initiating speech more quickly, but that advantage might only be revealed if the speaker has a high degree of cognitive speed. The individual cognitive resources that each speaker brings to bear on the task do seem to matter, but they do so only insofar as the language being spoken provides an avenue to exploit particular resources. For French speakers in the present task, in adopting a more incremental strategy, faster cognitive processing provided an advantage. Indeed, it was the French speakers with the highest levels of speed of processing that seemed most likely to adopt exactly that strategy. French speakers with high levels of processing speed were more likely to initiate their speech earlier, and were also less likely to gaze at the third picture of a display prior to speech onset.

One question that is important to address is why it might be that in French, unlike in English and German, participants planned more incrementally, and were thereby presented with an avenue to exploit the speed of cognitive processing. There are at least a few possibilities. One possibility raised by one of the reviewers of this manuscript is that post-nominal modification invites the most incremental planning strategies independently of the language. French speakers only modified N1 post-nominally. English and German speakers, on the other hand, produced a mixture: They sometimes modified N1 pre-nominally, and sometimes did so post-nominally (see Table 1). Hence, it could be that English and German speakers planned more incrementally when they produced post-nominal modifications of N1, and planned less incrementally when they modified pre-nominally. To explore this possibility, we examined the descriptive statistics for our three primary measures in the study—initiation times, pause frequencies, and gaze behaviors toward Region 3 during the initiation time window (see Table 3), broken down by language, display condition, and whether the first noun was modified pre-nominally, post-nominally, or not at all. Inspection of the table shows a pattern that is incompatible with the possibility that it was post-nominal modification by itself that invited more incremental strategies. Namely, for English and German, there is no evidence that it was pre-nominal modifications that accounted for the differences with French in these measures. In fact, inspecting the means, it appears that post-nominal modifications gave English and German speakers a higher load to plan up front (i.e., higher initiation times, more pauses during articulation). In a *post-hoc* analysis of only English and German utterances in which N1 was modified post-nominally, we did not find an association with speed of processing for any of our dependent variables. Hence, we do not find it likely that post-nominal modification provided a general benefit for speakers with high speed of processing to plan more incrementally—that effect only held for French.

**Table 3:** Descriptive statistics (means and standard deviations) for the three dependent variables, by language, display type (contrast vs. control), and N1 modification type.

	**Contrast display**			**Control display**		
	**Pre-nominal N1 modifier**	**Post-nominal N2 modifier**	**No N1 modifier**	**Pre-nominal N1 modifier**	**Post-nominal N2 modifier**	**No N1 modifier**
	***M* (*SD*)**	***M* (*SD*)**	***M* (*SD*)**	***M* (*SD*)**	***M* (*SD*)**	***M* (*SD*)**
**Initiation time in seconds**						
English	2.72 (1.88)	4.05 (4.30)	2.05 (1.83)	1.60 (0.81)	NA	1.60 (0.54)
French	NA	2.62 (2.03)	1.55 (0.93)	NA	1.12 (0.17)	1.46 (0.54)
German	3.28 (2.42)	4.23 (3.12)	1.90 (0.93)	NA	NA	1.78 (0.67)
**Number of pauses**						
English	2.74 (1.78)	3.11 (1.94)	2.89 (1.73)	2.00 (1.31)	NA	1.43 (1.11)
French	NA	3.85 (2.48)	4.35 (2.72)	NA	NA	2.44 (1.54)
German	1.83 (1.50)	2.18 (2.12)	3.43 (2.45)	NA	NA	1.04 (1.02)
**Proportion of initiation time gazing at region 3**						
English	0.23 (0.13)	0.26 (0.15)	0.15 (0.17)	0 (0)	NA	0.02 (0.06)
French	NA	0.25 (0.14)	0.07 (0.13)	NA	0 (0)	0.02 (0.07)
German	0.24 (0.10)	0.29 (0.13)	0.07 (0.15)	NA	NA	0.04 (0.07)

A second possible explanation of these cross-linguistic differences in planning strategies is that French speakers planned less in advance, and exploited cognitive speed, not simply because they could modify post-nominally, but because they faced no other choice. Although French speakers in the present task could have felt relatively comfortable adopting a planning strategy by which they would fixate the first object to be described in their utterance, immediately begin planning that noun, then incrementally plan any necessary modifications of that noun after speech onset has already begun, they also never faced the possibility of a pre-nominal modification. German and English speakers, on the other hand, faced that possible choice on every trial of the study. For these reasons, if there is a syntactic explanation for the differences, it is not simply that post-nominal modifications elicit more incremental strategies (e.g., Brown-Schmidt and Konopka, 2008), but rather that a greater variety of syntactic choices may elicit a less incremental strategy (e.g., Myachykov et al., 2013).

Although our results are consistent with this syntactic flexibility account, it would be pre-mature to draw that connection explicitly. There may, in fact, be other differences that exist between these languages that account for the differences in planning strategies beyond the present word order constraints. In particular, a prosodic difference exists in the phrasing properties of English/German vs. French. Phrasing refers to the grouping of words into larger units. English and German have Intonation Phrases and intermediate phrases, whereas French includes the Intonational Phrase and, at a lower level, a constituent which is posited between the Intonational Phrase and the word, the Accentual Phrase. Importantly for our study, the Accentual Phrase, the basic unit of French prosody, is generally smaller (containing fewer words) than the intermediate phrase (Jun and Fougeron, 2000). As prosodic phrases have been suggested to be used as potential planning units, specifically determining the chunk of the upcoming utterance to be encoded phonologically (Keating and Shattuck-Hufnagel, 2002; Krivokapić, 2007, 2012; Bishop, 2020; Bishop and Intlekofer, 2020), this raises the possibility that French might have smaller processing units than English and German, and thus French might be planned in smaller chunks (more incrementally) than English and German. Future research might investigate the extent to which these prosodic differences (or still other differences) among languages could more generally account for these cross-linguistic planning differences. Still, the important finding remains: When a language (French) was planned more incrementally in the present study, regardless of why it was planned more incrementally, it was associated with speed of processing in a way that less incrementally planned languages (English and German) were not.

Before concluding, it is important to address some limitations and other issues of the present research. One such issue is the fact that we did not find any evidence for statistically significant individual differences in the planning scope of English and German. Although German seemed to pattern largely along with English in initiation time, pause, and eye movement data, the effects of WM observed in Swets et al. (2014) in English did not replicate with statistical significance in English, nor did the effect emerge in German (although the overall trend looks similar for both languages). It is of course possible that these are true null effects. Another possible explanation for the lack of these effects is that the present study was underpowered to detect them. It is also possible that we did not obtain a significant effect for those languages in the present study due to differences in how we obtained samples. In the Swets et al. (2014) study that the present studies are meant to partially replicate and extend, the researchers used a two-phase data collection process. In Phase I, they first collected WM data from a large (*N* = 92) sample of participants. Then, to ensure a wide range of WM scores in the speech planning task, they selectively recruited 26 participants for Phase II, which was the language production task itself. The intent was to allow for the collection of labor-intensive speech production data from a smaller sample (*N* = 26) while still exploiting a fuller range of individual differences scores than would likely occur by chance in a sample of that size. In the present study, it was not possible to recruit the French or German participants in that manner, nor was it possible to recruit a sample larger than about 30 due to the hand-coding requirements of the language production data. Including all 93 participants from the three samples in the overall statistical model helped to reduce the problems associated with these smaller, more restricted samples, but when looking at each language individually, it is possible that we did not detect differences that would have been present had it been possible to collect data according to the method carried out in Swets et al. (2014). Because of the possibility that the overall study was underpowered, and to rule out the possibility of spurious effects, it will be important in future research to show that the relationship between speed of processing and planning scope that we did find in French can be more robustly supported.

More generally, one requisite caveat to add is that, because language, reading span scores, and letter comparison scores are all subject variables, and because any finding concerning any of those variables is correlational in nature, we acknowledge that any effect reported regarding those variables cannot be unambiguously interpreted in a directional, causal manner. For example, our language variable is not only a subject variable, but given that this is an early and exploratory attempt to study the interface of cross-linguistic and individual differences in the scope of speech planning, it is also not controlled to account for the many differences that exist between the languages. Although the three languages differ most obviously in terms of modifier word order, we have made no attempt to control for phonological, word length, speech rate, prosodic, or other grammatical differences that may exist among the three languages. But again, what matters most for the present study is that among the different languages examined, different cognitive factors were predictive of the scope of planning.

Regarding our cognitive predictor factors, although we favor the interpretation that planning processes are more likely to rely on external cognitive capacities such as WM and speed of processing, it is possible that there are un-measured, third variables that could more generally explain such relationships (such as general intelligence or exposure to language). It is also possible that the direction of the relationship between language spoken and cognitive capacity operates in the opposite direction from what we suppose. Along these lines, a recent paper by Amici et al. (2019) offers evidence that the mastery of a particular language “exercises” certain cognitive capacities more than other languages by virtue of having different word order requirements. In that study, the researchers conducted short term memory and WM tests among speakers of consistently left-branching languages (i.e., languages that require pre-nominal modification) and consistently right-branching languages (i.e., languages that require post-nominal modification). The results showed that speakers of left-branching languages performed better on items that appeared early in serial order in a WM task than items that appeared later, while speakers of right-branching languages showed the opposite effect. It is possible that a similar direction of influence could be occurring in our data, but we did not set out to test that possibility, and there is little in the data that we collected to suggest it as a possibility. It is also important to note that the Amici et al. (2019) study itself is correlational in nature, and is therefore not able to rule out other explanations for the results. This research, along with recent theoretical approaches to suggest that WM is reduceable to, or emergent from, the process of speech planning itself (Acheson and MacDonald, 2009; Acheson et al., 2011; Schwering and MacDonald, 2020)—suggest that the directionality of the relationship between cognitive capacities and speech planning is another important area for future research.

In conclusion, this study is the first that we are aware of to demonstrate that individual differences in cognitive capacities may vary in the degree to which they predict the scope of planning among speakers of different languages. It also shows that WM and speed of processing have unique contributions to the process of advance speech planning.

## Data Availability Statement

The raw data supporting the conclusions of this article will be made available by the authors, without undue reservation.

## Ethics Statement

Each study was conducted with approval from the appropriate bodies that oversee protections of human subjects at each site (Studies 1 and 2), or appropriately complied with the Declaration of Helsinki (Study 3). Study 1 was conducted with approval from the Institutional Review Board of Grand Valley State University, Study 2 with approval from the board of directors at ZAS. The participants provided their written informed consent to participate in these studies.

## Author Contributions

BS, SF, JK, and CP contributed to conception, design of the study, and wrote sections of the manuscript. BS, SF, and CP coordinated the collection of data. SF processed and organized the database. CP and SF performed the statistical analysis. BS wrote the first draft of the manuscript. All authors contributed to manuscript revision, read, and approved the submitted version.

## Conflict of Interest

The authors declare that the research was conducted in the absence of any commercial or financial relationships that could be construed as a potential conflict of interest.
