# Supplementary material for: A Cross-Linguistic Study of Individual Differences in Speech Planning
Source: Front Psychol. 2021 May 7;12:655516. doi: 10.3389/fpsyg.2021.655516 (PMC8139632; doi:10.3389/fpsyg.2021.655516)
Supplement: Supplementary file 1 [file Data_Sheet_1.docx]

**APPENDIX**

Note:

WM: working memory as indexed by mean-centered reading span scores

Speed: speed of processing as indexed by mean-centered letter comparison scores

**PRENOMINAL N1 MODIFICATION**

|  | Estimate | SE | *z* | *p* |
| --- | --- | --- | --- | --- |
| (Intercept) | -1.20433 | 0.77735 | -1.549 | 0.121 |
| WM | 0.05162 | 0.06773 | 0.762 | 0.446 |
| Speed | 0.11405 | 0.18427 | 0.619 | 0.536 |
| LanguageFrench | -7.86193 | 1.56540 | -5.022 | 5.11e-07 *** |
| LanguageGerman | -0.58548 | 0.68645 | -0.853 | 0.394 |
| WM:LanguageFrench | 0.11824 | 0.34335 | 0.344 | 0.731 |
| WM:LanguageGerman | -0.08074 | 0.09108 | -0.886 | 0.375 |
| Speed:LanguageFrench | -0.13349 | 0.61225 | -0.218 | 0.827 |
| Speed:LanguageGerman | 0.25973 | 0.28598 | 0.908 | 0.364 |

| **SPEECH ONSET TIME** |  |  | |  | | |  | |  | | |  |
| --- | --- | --- | --- | --- | --- | --- | --- | --- | --- | --- | --- | --- |
|  | | | Estimate | | SE | df | | *t* | | *p* |  | |
| (Intercept) | | | 8.308e-01 | | 7.553e-02 | 1.410e+02 | | 10.999 | | < 2e-16 | *** | |
| Display | | | -4.738e-01 | | 7.792e-02 | 7.341e+01 | | -6.080 | | 4.93e-08 | *** | |
| WM | | | 4.117e-03 | | 1.029e-02 | 1.272e+02 | | 0.400 | | 0.6897 |  | |
| Speed | | | 2.643e-02 | | 2.811e-02 | 1.296e+02 | | 0.940 | | 0.3489 |  | |
| LanguageFrench | | | -2.337e-01 | | 9.147e-02 | 1.347e+02 | | -2.555 | | 0.0117 | * | |
| LanguageGerman | | | 1.622e-01 | | 9.155e-02 | 1.334e+02 | | 1.772 | | 0.0787 | . | |
| Display:WM | | | -3.957e-03 | | 9.305e-03 | 8.614e+01 | | -0.425 | | 0.6717 |  | |
| Display:Speed | | | -2.446e-02 | | 2.531e-02 | 8.679e+01 | | -0.966 | | 0.3365 |  | |
| Display:LanguageFrench | | | 1.794e-01 | | 7.862e-02 | 8.391e+01 | | 2.282 | | 0.0250 | * | |
| Display:LanguageGerman | | | 7.367e-04 | | 7.872e-02 | 8.304e+01 | | 0.009 | | 0.9926 |  | |
| WM:LanguageFrench | | | -7.232e-03 | | 1.646e-02 | 1.272e+02 | | -0.439 | | 0.6611 |  | |
| WM:LanguageGerman | | | -3.802e-03 | | 1.392e-02 | 1.244e+02 | | -0.273 | | 0.7852 |  | |
| Speed:LanguageFrench | | | -1.023e-01 | | 4.150e-02 | 1.252e+02 | | -2.464 | | 0.0151 | * | |
| Speed:LanguageGerman | | | -3.788e-02 | | 4.248e-02 | 1.246e+02 | | -0.892 | | 0.3742 |  | |
| Display:WM:LanguageFrench | | | 6.944e-03 | | 1.482e-02 | 8.621e+01 | | 0.469 | | 0.6405 |  | |
| Display:WM:LanguageGerman | | | 4.176e-03 | | 1.253e-02 | 8.434e+01 | | 0.333 | | 0.7398 |  | |
| Display:Speed:LanguageFrench | | | 5.087e-02 | | 3.732e-02 | 8.415e+01 | | 1.363 | | 0.1765 |  | |
| Display:Speed:LanguageGerman | | | 7.751e-03 | | 3.823e-02 | 8.400e+01 | | 0.203 | | 0.8398 |  | |

**POSTNOMINAL N1 MODIFICATION**

|  | Estimate | SE | *z* | *p* |  |
| --- | --- | --- | --- | --- | --- |
| (Intercept) | -1.48344 | 0.57163 | -2.595 | 0.00946 | ** |
| WM | -0.02561 | 0.05887 | -0.435 | 0.66358 |  |
| Speed | 0.07089 | 0.16804 | 0.422 | 0.67312 |  |
| LanguageFrench | 2.00773 | 0.77559 | 2.589 | 0.00964 | ** |
| LanguageGerman | 0.30105 | 0.77577 | 0.388 | 0.69796 |  |
| WM:LanguageFr | 0.12636 | 0.09338 | 1.353 | 0.17598 |  |
| WM:LanguageGer | 0.09078 | 0.07942 | 1.143 | 0.25304 |  |
| SpeedSpeed:LanguageFrench | -0.34457 | 0.23719 | -1.453 | 0.14629 |  |
| Speed:LanguageGerman | -0.16430 | 0.24300 | -0.676 | 0.49897 |  |

**NUMBER OF PAUSES**

|  | Estimate | SE | *z* | *p* |  |
| --- | --- | --- | --- | --- | --- |
| (Intercept) | 1.051307 | 0.097079 | 10.829 | < 2e-16 | *** |
| Display | -0.733265 | 0.100270 | -7.313 | 2.61e-13 | *** |
| WM | -0.017639 | 0.013589 | -1.298 | 0.1943 |  |
| Speed | 0.019147 | 0.039042 | 0.490 | 0.6238 |  |
| LanguageFrench | 0.274545 | 0.121994 | 2.250 | 0.0244 | * |
| LanguageGerman | -0.271710 | 0.124123 | -2.189 | 0.0286 | * |
| Display:WM | 0.001083 | 0.013516 | 0.080 | 0.9361 |  |
| Display:Speed | -0.049192 | 0.037945 | -1.296 | 0.1948 |  |
| Display:LanguageFrench | 0.195352 | 0.107000 | 1.826 | 0.0679 | . |
| Display:LanguageGerman | -0.111183 | 0.119742 | -0.929 | 0.3531 |  |
| WM:LanguageFrench | 0.008546 | 0.021316 | 0.401 | 0.6885 |  |
| WM:LanguageGerman | -0.011374 | 0.018355 | -0.620 | 0.5355 |  |
| Speed:LanguageFrench | -0.089467 | 0.057345 | -1.560 | 0.1187 |  |
| Speed:LanguageGerman | -0.044443 | 0.064072 | -0.694 | 0.4879 |  |
| Display:WM:LanguageFrench | -0.011496 | 0.019719 | -0.583 | 0.5599 |  |
| Display:WM:LanguageGerman | 0.019454 | 0.018909 | 1.029 | 0.3036 |  |
| Display:Speed:LanguageFrench | 0.084744 | 0.051427 | 1.648 | 0.0994 | . |
| Display:Speed:LanguageGerman | 0.021022 | 0.057583 | 0.365 | 0.7151 |  |

**GAZE TIME**

|  | Estimate | SE | z | p |  |
| --- | --- | --- | --- | --- | --- |
| (Intercept) | -1.77946 | 0.25140 | -7.078 | 1.46e-12 | *** |
| Display | -3.44572 | 0.36378 | -9.472 | < 2e-16 | *** |
| WM | 0.05947 | 0.03746 | 1.588 | 0.1123 |  |
| Speed | 0.04457 | 0.10248 | 0.435 | 0.6636 |  |
| LanguageFrench | -0.51241 | 0.32754 | -1.564 | 0.1177 |  |
| LanguageGerman | 0.10025 | 0.32356 | 0.310 | 0.7567 |  |
| Display:WM | -0.04983 | 0.05366 | -0.929 | 0.3531 |  |
| Display:Speed | -0.01775 | 0.14790 | -0.120 | 0.9045 |  |
| Display:LanguageFrench | 0.38378 | 0.46675 | 0.822 | 0.4110 |  |
| Display:LanguageGerman | 0.48759 | 0.44916 | 1.086 | 0.2777 |  |
| WM:LanguageFrench | -0.03815 | 0.06054 | -0.630 | 0.5287 |  |
| WM:LanguageGerman | -0.03235 | 0.05067 | -0.638 | 0.5232 |  |
| Speed:LanguageFrench | -0.32935 | 0.15433 | -2.134 | 0.0328 | * |
| Speed:LanguageGerman | 0.01838 | 0.15505 | 0.119 | 0.9056 |  |
| Display:WM:LanguageFrench | 0.06988 | 0.08733 | 0.800 | 0.4236 |  |
| Display:WM:LanguageGerman | 0.07272 | 0.07071 | 1.028 | 0.3038 |  |
| Display:Speed:LanguageFrench | 0.31138 | 0.22851 | 1.363 | 0.1730 |  |
| Display:Speed:LanguageGerman | -0.17135 | 0.21841 | -0.785 | 0.4327 |  |
